# An intelligent framework for crop health surveillance and disease management

**DOI:** 10.1371/journal.pone.0324347

**Published:** 2025-05-23

**Authors:** Yasser M. Ayid, Yasser Fouad, Mourad Kaddes, Heba M. El-Hoseny

**Affiliations:** 1 Mathematics Department, Applied Collage Al-Kamil Branch, University of Jeddah, Jeddah, Saudi Arabia; 2 Department of Computer Science, Faculty of Computers and Information, Suez University, Suez, Egypt; 3 Department of Information Systems, College of Computing & Information Technology at Khulais, University of Jeddah, Jeddah, Saudi Arabia; 4 Faculty of Computer Studies, Arab Open University, Riyadh, Saudi Arabia; Sri Padmavati Mahila Visvavidyalayam, INDIA

## Abstract

The agricultural sector faces critical challenges, including significant crop losses due to undetected plant diseases, inefficient monitoring systems, and delays in disease management, all of which threaten food security worldwide. Traditional approaches to disease detection are often labor-intensive, time-consuming, and prone to errors, making early intervention difficult. This paper proposes an intelligent framework for automated crop health monitoring and early disease detection to overcome these limitations. The system leverages deep learning, cloud computing, embedded devices, and the Internet of Things (IoT) to provide real-time insights into plant health over large agricultural areas. The primary goal is to enhance early detection accuracy and recommend effective disease management strategies, including crop rotation and targeted treatment. Additionally, environmental parameters such as temperature, humidity, and water levels are continuously monitored to aid in informed decision-making. The proposed framework incorporates Convolutional Neural Network (CNN), MobileNet-1, MobileNet-2, Residual Network (ResNet-50), and ResNet-50 with InceptionV3 to ensure precise disease identification and improved agricultural productivity.

## I. Introduction

Agriculture is vital to global food security and economic stability, but plant diseases reduce crop output and quality. Maintaining agricultural sustainability requires early and precise disease diagnosis. New research in AI technology and deep learning variant techniques have shown significant promise in automating the identification approaches of plant diseases, facilitating prompt responses, and minimizing the utilization of harmful chemicals [1].

Farmers and agronomists have always relied on hands-on examinations to detect plant diseases. This method consumes a lot of time and is also lead to mistakes, frequently resulting in late detection, which in turn leads to more usage of pesticides, a decrease in crops, and economic losses. AI-based disease detection allows sustainable farming with less chemical use [2].

Smart agriculture is increasingly applying technology to achieve sustainability, boost productivity, and makes less costs. In smart irrigation, a scheduling tool has been proposed based on cloud computing technologies [2–4]. By tracking and analyzing environmental options, farmers can enhance practices, make valuable decisions regarding usage of resources, and detect yield issues at the near stage. This technique enhances sustainability and fosters a deeper understanding of the relationship between farming and the land [1]. Smart diagnosis for plant diseases leverages new technologies like artificial intelligence, machine learning, and image recognition to quickly and accurately identify plant health issues. These systems process and handle data from sensors, cameras, and other tools to detect diseases early, enabling farmers to take timely action. By automating diagnosis, smart systems reduce reliance on manual inspection, improve crop management, and improve sustainability by minimizing the use of chemicals and resources. This technology is revolutionizing agricultural practices, making them more efficient and precise [5].

Different researchers have introduced solutions for plant disease detection to improve crop productivity and enhance food security. Araujo et al. [6] developed a technique for plant diagnosis based on an enhanced feature-extracting Support Vector Machine (SVM) using a Bag of Visual Words (BoVW) and Local Binary Patterns (LBPs) methods that are used for processing as well as feature extraction. Sh. and P. [7] introduced a rice disease classification algorithm based on color features and SVM classifier. A total of 172 different color channels were implemented for feature extraction classified four different types of rice diseases and achieved 94.68% accuracy.

Chowdhury et al. [8] employ a deep learning architecture with EfficientNet to categorize tomato illnesses in 18,161 tomato leaf pictures. The effectiveness of the two segmentation models, Modified U-net and U-net, is compared for binary, six-class, and ten-class classifications. With remarkable accuracy, Intersection over Union (IoU), and Dice scores of 98.66%, 98.5%, and 98.73%, respectively, the improved U-net model was achieved. EfficientNet-B7 demonstrated exceptional performance in binary and six-class classifications, with accuracies of 99.95% and 99.12%, respectively. The experimental experiments surpass the current literature.

Sharma et al. [9] introduced a CNN model for classifying illnesses affecting rice and potato plant leaves. The model, trained on datasets of 5,932 and 1,500 photos, attained an accuracy of 99.58% for rice images and 97.66% for potato leaves. It outperformed several machine learning image classifiers, such as Random Forest, K-Nearest Neighbors, Decision Tree, and Support Vector Machine.

Computer-based program developed by Haridasan et al. [10] to identify and diagnose rice plant illnesses from photos. This system that was developed by them employs image processing, machine learning, and deep learning methodologies to detect and classify infection based on visual data. SVM classifiers and convolutional neural networks diagnose disease. Deep learning-based strategies had the greatest validation accuracy of 0.9145, and a predictive cure is advised for agriculture-related companies and individuals.

S and H [11] used photos of tomato leaves along with six diseases and healthy samples to diagnose tomato plant leaf illnesses using Fuzzy-SVM, CNN, and Region-based Convolutional Neural Network (R-CNN). The type of disease is identified using R-CNN classifiers. Fuzzy SVM and CNN classification algorithms are evaluated to determine which plant disease prediction model produces the best results; the R-CNN-based classifier achieves the maximum accuracy, measuring 96.735%.

In smart agriculture, IoT-based fertilizer management is a key application that leverages IoT technology to optimize fertilizer usage. This system provides farmers with real-time data on soil conditions and nutrient levels, enabling them to adjust the type and amount of fertilizer applied to their crops for more efficient and targeted fertilization [8,9].

Agriculture faces multifaceted challenges that hinder its sustainability and productivity [10]. These challenges include climate change-induced uncertainty, water scarcity, soil degradation, pest and disease outbreaks, and limited technology access. Addressing these issues is crucial for ensuring food security, supporting rural livelihoods, and promoting sustainable farming practices. Effective solutions involve climate-resilient agriculture, improved water management, soil conservation, disease-resistant crop varieties, and technology dissemination to empower farmers. Additionally, supportive policies and investments in infrastructure are essential to tackle these challenges effectively.

This paper presents a smart agricultural system that manages the farming process, including crop monitoring, fertilizer administration, climate condition monitoring, and automatic irrigation. The system, powered by a Node Microcontroller Unit (Node-MCU), includes sensors like DHT11, Moisture, LDR, Water Pump, and 12V LED strip. The IoT-based system collects data on soil moisture, temperature, humidity, and temperature, sending it to a cloud for live monitoring. This system optimizes resource utilization, increases productivity, and improves decision-making in farming operations. Critical data on weather, soil moisture, temperature, humidity, and other environmental parameters is gathered via IoT sensors. The data is stored, processed, and analyzed on a centralized cloud platform. The mobile application allows farmers to remotely access the system, adjust irrigation settings, monitor sensor readings, receive alerts, and automate processes. This integration brings benefits such as precise resource management, reduced water consumption, fertilizer usage, and reduced environmental impact. Table 1 displays some related studies to the proposed work.

**Table 1:** Some related studies to the proposed work.

Ref.	Methodology	Contribution	Accuracy	Limitation
Araujo et al. [6]	SVM with BoVW and LBPs for plant diagnosis	BoVW, LBPs, SVM for feature extraction and diagnosis	75.8%	Low accuracy, traditional feature extraction, limited generalization
Sh. and P. [7]	Classify Rice leaves diseases using SVM and 172 color channels	Color features and SVM; 172 color channels	94.68%	Relies only on color features, which can be affected by lighting conditions
Chowdhury et al. [8]	Deep learning with EfficientNet for tomato disease classification	EfficientNet-B7, U-net; high accuracy in binary and multiclass	98.66% (binary), 99.95% (EfficientNet-B7)	Requires high computational power, limited to specific crops
Sharma et al. [9]	CNN model for rice and potato leaf disease classification	CNN; comparison with SVM, KNN, Decision Tree, Random Forest	99.58% (rice), 97.66% (potato)	No explainability in the decision-making process
Haridasan et al. [10]	Image processing, Machine Learning, and Deep Learning for rice plant disease identification	SVM, CNN; validation accuracy of 0.9145	0.9145	Limited dataset, potential overfitting
S and H [11]	Fuzzy-SVM, CNN, R-CNN for tomato disease classification	Fuzzy-SVM, CNN, R-CNN; highest accuracy of 96.735%	96.735	Computational complexity, high training time

The proposed framework addresses the limitations of previous studies, which mainly focus on image-based plant disease classification without considering environmental factors. In contrast, this system integrates real-time monitoring of temperature, soil moisture, and plant health, providing instant alerts for early intervention. To enhance model generalization, the framework has been trained on a large dataset of 87,000 images across 38 plant classes from Kaggle, ensuring higher accuracy across diverse conditions. Unlike prior approaches that lack decision support, this framework not only detects diseases using deep learning but also offers personalized recommendations for pesticides, fertilizers, and optimal farming practices. This makes it a more comprehensive, efficient, and practical solution for modern agriculture.

The main contribution is the proposal of an intelligent framework rather than just a mobile application that offers a variety of services designed to assist farmers in improving crop management.

The system continuously monitors key environmental parameters, such as temperature, soil humidity, and plant health, while providing real-time alerts about any significant changes or emergencies. This allows farmers to respond promptly to issues like plant diseases, extreme weather, or soil conditions. Additionally, the framework employs deep learning techniques for automatic plant disease detection, enabling accurate identification of various diseases. This helps farmers quickly diagnose plant health issues and take appropriate action, thus minimizing crop damage and enhancing overall productivity.

Moreover, the framework provides tailored recommendations to improve plant health, such as suggesting suitable pesticides for different plant diseases or advising on fertilizers that enhance crop growth. The system also enables continuous monitoring empowering farmers to make data-driven decisions to maximize crop yield. By incorporating Deep learning algorithms, the system’s recommendations become more precise over time, offering even better guidance.

By automating much of the monitoring and decision-making process, the framework reduces manual effort and increases agricultural efficiency. This intelligent framework provides a comprehensive solution that addresses several critical aspects of modern farming, simplifying the process and improving productivity.

The primary motivations for proposing this framework include the need for efficient crop management through continuous monitoring and real-time data, the importance of early detection of plant diseases using deep learning techniques to provide faster and more accurate diagnoses, and the goal of improving agricultural productivity by offering tailored recommendations for pesticides and fertilizers. Additionally, the framework aims to reduce manual labor by automating various farming processes, thus enabling farmers to make data-driven decisions for enhanced crop management.

This paper’s remaining sections are organized as follows. In Preliminaries section, the IoT-based agricultural systems preliminary aspects are examined and clarified. The suggested framework, including mobile application and deployment, is examined in the proposed algorithm and model deployment sections. The assessment metrics analysis and its numerical outcomes are examined in evaluation metrics section. The simulation analysis is examined and discussed in simulations and discussions section. The conclusions section concludes the paper.

## II. Preliminaries

### 1. Agriculture systems powered by IoT

**Fig 1:**
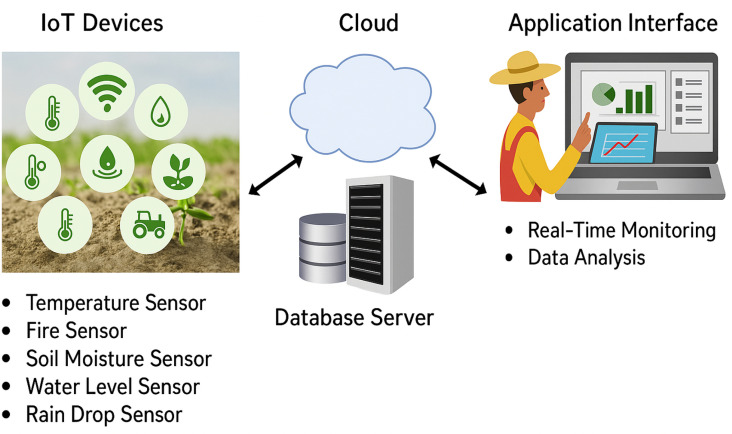
The smart agriculture system’s IoT architecture.

To improve farming techniques and boost productivity, IoT-based agricultural systems make use of a variety of cutting-edge frameworks and technology. Fig 1 demonstrates the IoT architecture for the smart farm system. IoT-based agricultural systems incorporate a wide range of technologies and frameworks, and A brief overview of them can be introduced [12–17].

**Wireless Sensor Networks:** low-cost, low-cost sensor nodes that monitor environmental factors that impact on industry like temperature, humidity, soil moisture, and light intensity, providing real-time data for agriculture decision-making [1,13].

**IoT Platforms:** IoT platforms, such as Microsoft Azure IoT, AWS IoT, Google Cloud IoT, and IBM Watson IoT, are essential for managing and connecting IoT devices and data, offering features like device management, data visualization, analytics, and integration [14–17].

**Edge Computing:** Edge computing in agricultural IoT systems allows on-time data processing and analysis at the field level, minimizing latency and bandwidth usage, quicker decision-making, and reducing reliance on cloud resources.

**Artificial intelligence (AI) and machine learning (ML):** These two fields use sensor data to provide insights and forecasts that help with pest and disease diagnosis, yield prediction, and resource management for fertilizer and water use.

**Geographic Information Systems:** combines spatial and agricultural data, enabling farmers to make location-based decisions for soil mapping, agronomic monitoring, and high-tech farming.

**Drones and UAVs:** UAVs, equipped with cameras and sensors, are utilized for aerial imaging, crop monitoring, surveillance, and providing high-resolution data for crop health assessment and yield estimation.

**Smart Irrigation Systems:** IoT-enabled smart irrigation systems employ sensors to assess soil moisture and meteorological conditions, allowing accurate and efficient watering, water saving, and enhancement of agricultural yields [18].

**Reduced-Power Wide-Area Networks:** depicts technologies such as LoRaWAN and NB-IoT have extensive communication capabilities with little power usage, rendering them suitable for linking IoT devices in isolated agricultural regions.

**Open-source frameworks and Libraries:** Arduino, Raspberry Pi, TensorFlow, and OpenCV enable developers to create customized IoT solutions for agricultural needs.

IoT-based agricultural systems enable farmers with real-time insights, automation, and information-driven choices by integrating various technologies and frameworks, which eventually enhance farming operations’ efficiency, sustainability, and profitability [19,20].

### 2. Crop monitoring IoT systems

**Fig 2:**
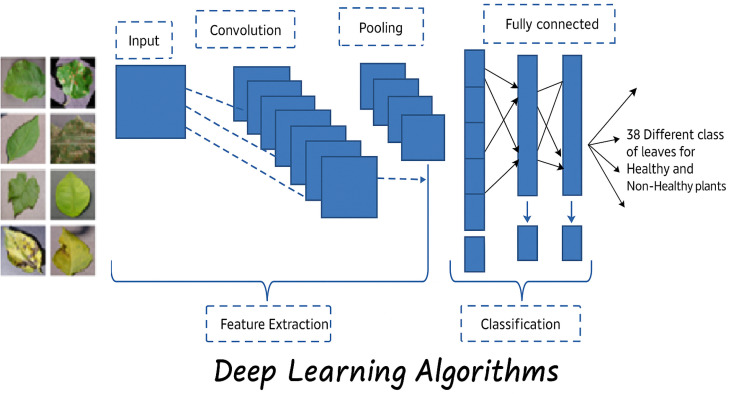
The general framework for plant disease classification approach.

Crop monitoring IoT systems are crucial for modern agriculture, offering real-time insights into crop health, growth conditions, and environmental factors [21–23]. Sensors continuously collect data on environmental conditions and crop parameters. This data includes temperature, humidity levels, soil moisture content, nutrient levels, and more. Sensor nodes use protocols such as Wi-Fi, Bluetooth, Zigbee, or LoRaWAN to establish wireless communication with a central hub or gateway. Over vast agricultural areas, data transmission is made possible with ease by this link. Either locally at the edge or remotely to the cloud, the acquired data is processed for analysis. To find trends, spot abnormalities, and forecast crop growth, insect infestations, or the spread of diseases, machine learning algorithms can examine the data. Farmers can access the processed data through web or mobile applications. Visualization tools present the data in easy-to-understand formats such as charts, graphs, and heatmaps [23,24]. These insights help farmers make informed decisions about irrigation scheduling, fertilization, pest control, and harvesting [25–27]. The general framework for plant disease detection can be presented in Fig 2.

### 3. Convolutional neural network (CNN) architecture

Deep learning is an artificial intelligence neural network composed of several layers, analogous to the activities of the brain. It has been utilized in several domains, including social networking, e-commerce, and object identification. CNN, a multi-layered deep neural network, has demonstrated exceptional efficacy in data processing. It has two stages: feature picture extraction and classification [28–30].

The main idea behind CNNs is to take local features from higher layers and move them to lower layers to obtain more complex features. To minimize the dimensions of the input volume, the convolutional layer consists of a group of kernels that connect the entire input using “stride(s)” to find feature mappings. The convolutional layer computation equation is as follows [25]:


F(i,j)=(I*K\rightleft(i,j)=∑∑I(i+m,j+n)K(m,n) (1)

where (I*K) depicts the layer that performs the convolution operation, I is the input matrix, 2D filter K of m×n size and Fis a 2D feature output map. By maintaining the threshold input at zero, the ReLU layer computes activation and increases the nonlinearity of the feature maps which can be expressed mathematically as:


F(X)=max (0,X) (2)

The layer of pooling reduces image attributes while retaining important information through Max Pooling, average Pooling, and sum Pooling. Max Pooling is the most common type. The FC layer classifies feature outputs using activation functions like soft-max or sigmoid for final decision-making.

## III. The proposed algorithm

The Smart Agriculture System aspires to cater to a multitude of user and system requirements to deliver an effective and user-friendly solution. From the user perspective, the system must be easy to use, allowing farmers with various technical backgrounds to access its benefits effortlessly. Real-time monitoring capabilities are paramount to enable timely decisions regarding irrigation, nutrients, and disease control. Security and privacy are the most prevalent concerns, and the system must ensure data protection and comply with privacy regulations. Compatibility with major smartphone platforms, affordability, and accessibility even in regions with limited connectivity are expected features. Customization options empower users to tailor the system to their specific crops and farming practices.

**Fig 3:**
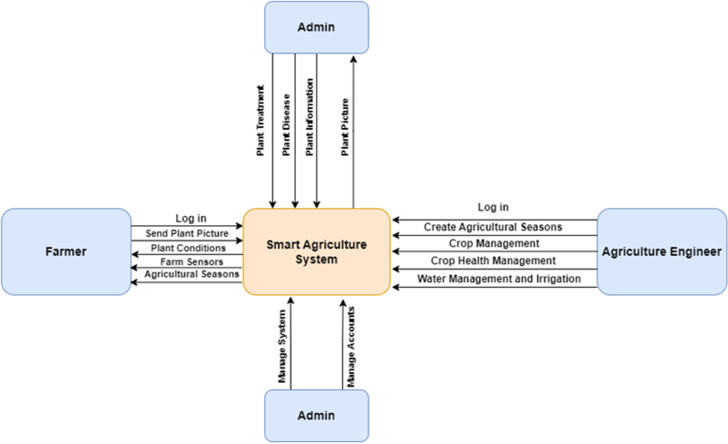
The main data flow diagram for the proposed framework and required tasks.

From the system’s viewpoint, scalability is a fundamental requirement to accommodate the project’s growth. Advanced data analytics capabilities are essential for processing sensor data, generating valuable insights, and offering recommendations. Reliability is a key aspect of minimizing downtime, while sufficient data storage, seamless integration with IoT sensors, and stringent data security measures are non-negotiable. Environmental compatibility and eco-friendliness are vital in today’s context. Technical support must be readily available to assist users, and the system should adhere to industry standards and regulations, including data privacy rules. In addition, the provision of comprehensive documentation and user manuals ensures users can easily set up, operate, and troubleshoot the system. The Smart Agriculture System is driven by a commitment to align these user and system requirements to develop a comprehensive solution that enhances the efficiency, sustainability, and productivity of the agriculture sector. Therefore, Fig 3 shows the major data flow diagram for the suggested model and associated tasks.

**Fig 4:**
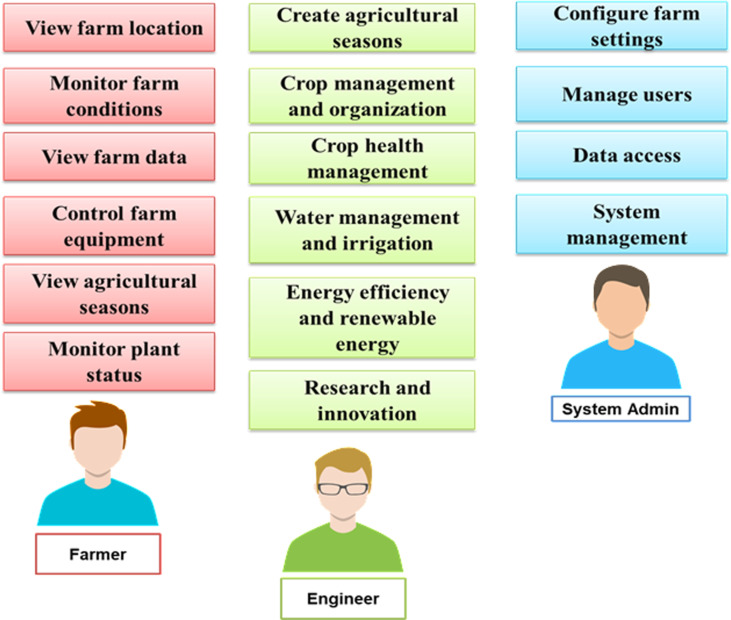
The Use case diagram for the implemented mobile application.

**Fig 5:**
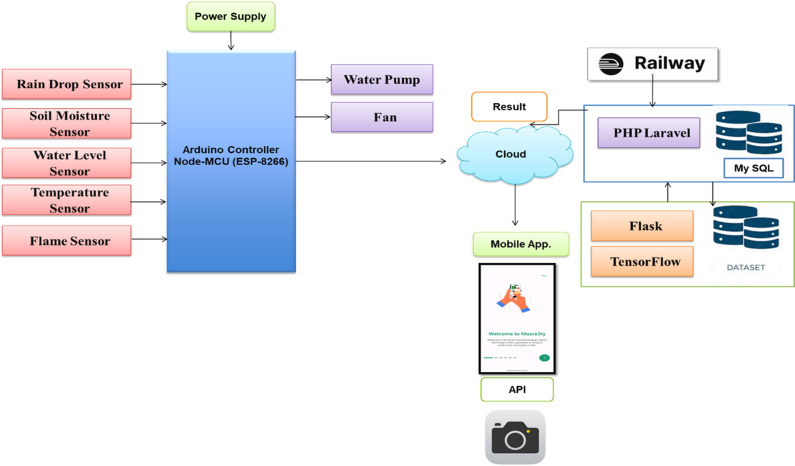
The main block diagram of the suggested framework.

The system employs n-tier architecture, a validated software model, to facilitate enterprise-level that run on clients and servers. It provides scalability, security, fault tolerance, reusability, and maintainability. The Use case of the proposed framework is introduced in Fig 4. Also, the main block diagram for the proposed framework is presented in Fig 5.

Below is the structured pseudo-code that outlines the core functionality of the proposed Smart Agriculture Framework:

**Initialize** Sensors, Database, and Deep Learning Model

**WHILE** the System is Running **DO**

** Read** Sensor Data (temperature, moisture, humidity)

** Capture** Plant Image

 Store Data

** IF** Abnormal Conditions are Detected **THEN**

**  Send** Alert to Farmer

** IF** Disease Found in Image, **THEN**

**  Identify** Disease and Suggest Treatment

** Provide** Recommendations (Irrigation, Fertilizer, Pesticide)

** Update** System with New Data

**END** WHILE

The pseudo-code defines input variables such as temperature, soil moisture, humidity, and plant images, and output variables such as alerts, disease identification, and recommended actions (irrigation, fertilization, or pesticide application). This is implemented using Python as the primary programming language, particularly for deep learning and backend processing. Additionally, Sensor data and image data are used separately to make decisions. Sensor data, such as temperature, humidity, and soil moisture, is collected and analyzed in real-time. If abnormal conditions like high temperature or low moisture are detected, alerts are sent to the farmer to take appropriate actions, such as irrigation. On the other hand, plant images are captured and processed using deep learning models to detect diseases. If a disease is identified, it is classified, and recommendations for treatment, such as pesticide application, are provided.

## IV. Evaluation metrics

The performance of the proposed model is assessed using different metrics such as accuracy, Boundary Recall, Precision, F measure, and Loss [29]. The assessment metrics are established utilizing formulae such as TP, TN, FP, and FN, which denote true positives, true negatives, false positives, and false negatives, respectively. True positives occur when the model accurately identifies the positive class, while false positives arise when the model erroneously predicts the negative class, and these variables are measured to calculate evaluation metrics as Equations 3–5.

The accuracy refers to the percentage of correctly classified image pixels (Xiao et al. 2018).


$$Accuracy=\frac{correctly\;⥂⥂\;predicted\;pixels}{Total\;⥂\;⥂\;image\;pixels}=\frac{TP+TN}{TP+FP+TN+FN}$$ (3)

Precision refers to the fraction of real positive predictions (properly identified positive cases) among all occurrences projected as positive. It helps to answer the question: How many of the positive examples categorized by the model were truly correct?


$$Precision=\frac{TP}{TP+FP}$$ (4)

Boundary Recall refers to the proportion of illness pixels in the ground truth that was successfully identified by automated segmentation. The sensitivity can be estimated by [27].


$$Sensitivity=\frac{correctly\;⥂⥂\;predicted\;disease\;\;pixels}{Total\;⥂\;⥂number\;of\;actual\;disease\;\;pixels}=\frac{TP}{TP+FN}$$ (5)

The F measure evaluates precision and recall, ensuring high values for segmented images align with ground truth in close locations. The F measure can be estimated as [30]:


$$\begin{array}{c} F_{measure}=2\times \frac{\;Precision\times Recall}{\;Precision⊕Recall} \end{array}$$ (6)

## V. Simulations and discussions

The proposed framework utilizes five deep learning algorithms for plant disease classification: MobileNet 1, MobileNet 2, ResNet 50, ResNet 50-Inception V3, and CNN. These models were chosen for their ability to process large-scale image datasets efficiently. MobileNet 1 and MobileNet 2 are lightweight architectures optimized for fast inference, making them ideal for real-time applications. ResNet 50 employs residual learning to facilitate deeper network training and improve accuracy. ResNet 50-Inception V3 integrates ResNet’s depth with Inception’s parallel convolutional layers, enhancing feature extraction. The CNN model serves as a baseline for comparison, offering a conventional deep-learning approach.

Training and evaluation were conducted using **87,000 images spanning 38 plant classes**, sourced from Kaggle for both healthy and non-healthy datasets [31]. Performance assessment was based on key metrics, including **accuracy, precision, recall, F1 score, and loss**, ensuring a comprehensive comparison of the models. The implemented technologies and tools that have been used to develop this framework are shown in Table 2. The trained dataset can be presented in Table 3. The subsequent section presents the detailed evaluation results.

**Table 2:** Used technologies and tools.

Software Requirements						Hardware Requirements
Front-end	Flutter App	Back-end	Database	Deep Learning	IDE	Hardware
Html-CSS JavaScript Bootstrap jQuery Typescript Angular 16	Dart Bloc State Management Firebase HTTP	Nodejs Postman Express JS	Mongoose	Resnet 50 Inception Net CNN	VS-Code Android Studio PyCharm Arduino IDE Jupyter Google Colab	Node-MCU (ESP-8266) Arduino UNO ESP-32 ESP-Cam Soil Moisture Water Level Water Pump DHT22 NPK Fan Rain Drop Flame Sensor Resistors Transistors Capacitors Jumper Cable Relay Module

**Table 3:** The trained dataset for 38 different classes of plant leaves.

Plant Type	Plant Class	Trained Dataset	Tested Dataset
Apple	Apple_scab	2016	504
	Black_rot	1987	497
	Cedar_apple_rust	1760	440
	healthy	2008	502
Blueberry	healthy	1816	454
Cherry_(including_sour)	healthy	1826	456
	Powdery_mildew	1683	421
Corn_(maize)	Cercospora_leaf_spot Gray_leaf_spot	1642	410
	Common_rust	1907	477
	healthy	1859	465
	Northern_Leaf_Blight	1908	477
Grape	Black_rot	1888	472
	Esca_(Black_Measles)	1920	480
	healthy	1692	423
	Leaf_blight_(Isariopsis_Leaf_Spot)	1722	430
Orange	Haunglongbing_(Citrus_greening)	2010	503
Peach	Bacterial_spot	1838	459
	healthy	1728	432
Pepper,_bell	Bacterial_spot	1913	478
	healthy	1988	497
Potato	Early_blight	1939	485
	healthy	1824	456
	Late_blight	1939	485
Raspberry	healthy	1781	445
Soybean	healthy	2022	505
Squash	Powdery_mildew	1736	434
Strawberry	healthy	1824	456
	Leaf_scorch	1774	444
Tomato	Bacterial_spot	1702	425
	Early_blight	1920	480
	healthy	1926	481
	Late_blight	1851	463
	Leaf_Mold	1882	470
	Septoria_leaf_spot	1745	436
	Spider_mites Two-spotted_spider_mite	1741	435
	Target_Spot	1827	457
	Tomato_mosaic_virus	1790	448
	Tomato_Yellow_Leaf_Curl_Virus	1961	490

**Fig 6:**
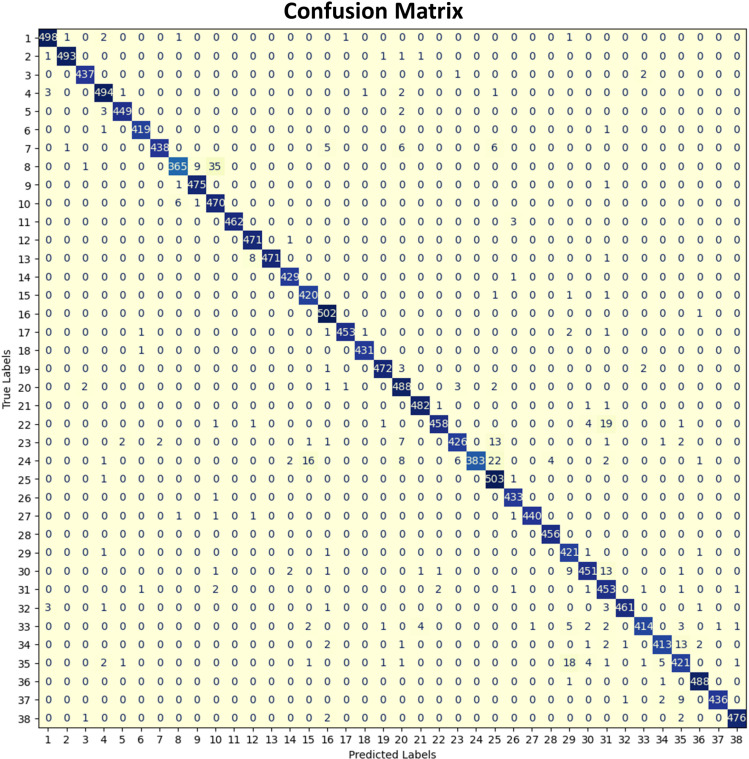
Confusion matrix for 38-class plant disease classification based on the proposed ResNet-50-InceptionV3 model.

In the proposed framework, five deep learning algorithms have been adopted to achieve the highest performance that can be practically implemented for such a large dataset. These algorithms are MobileNet 1, MobileNet 2, ResNet 50, ResNet 50- Inception V3, and CNN. The input datasets have been trained for all deep learning algorithms and evaluated based on different evaluation metrics such as accuracy, precision, recall, F1 score, and loss. From the obtained results, it has been shown that ResNet 50 with InceptionV3 has introduced the highest performance with the highest accuracy value of 92.9% and precision of 92.96%. The comparison results are introduced in Table 4. Also, the confusion matrix is presented in Fig 6.

**Table 4:** The performance evaluation for different deep learning depicted approaches.

Model	Accuracy	Precision	Recall	F1-Score	Loss
**MobileNet 1**	91.31%	91.36%	91.30%	91.39%	0.0014
**MobileNet 2**	92.12%	92.23%	92.25%	92.29%	1.32E-04
**ResNet 50**	92.85%	92.91%	92.85%	92.85%	1.88E-04
**ResNet 50 - InceptionV3**	**92.90%**	**92.96%**	**92.98%**	**92.97%**	**0.082**
**CNN**	90.23%	90.26%	90.13%	90.30%	6.67E-04

In addition to reporting performance metrics, we conducted statistical significance testing to validate the differences in model performance. A paired t-test between ResNet-50-InceptionV3 and CNN yielded a t-statistic of 13.90 and a p-value < 0.001, indicating that the performance difference is statistically significant. The 95% confidence interval of the mean difference was between 225.81% and 313.57%. Furthermore, a one-way ANOVA test across all models confirmed significant differences in their accuracies (F(4,45) = 137.06, p < 0.0001), as shown in Table 5.

**Table 5:** Statistical test results.

Test	Statistic	p-value	95% Confidence Interval
Paired t-test (ResNet-50-InceptionV3 vs CNN)	13.9036	0.0001	225.81% to 313.57%
One-way ANOVA (All Models)	137.0581	0.0001	

Hyperparameters are essential factors that influence the performance of a deep learning model, such as batch size and epochs. The proposed models have been trained using batch-size = 100 and epochs = 40 and evaluated using ROC curves to visualize the training process. The selection of hyperparameters is aimed at balancing model training efficiency and performance. A batch size of 100 strikes a balance between computational efficiency and model stability besides processing data without compromising the quality of model updates. Smaller batch sizes might slow down the training process but offer more frequent updates, while larger sizes might reduce update frequency and affect convergence.

Setting the number of epochs to 40 allows the model sufficient iterations to learn from the data without risking overfitting. Fewer epochs could lead to underfitting, while more could cause overfitting. With 40 epochs, the model is expected to learn effectively, especially when early stopping is used to prevent unnecessary training once performance plateaus.

The Adam optimizer is used due to its ability to automatically adjust learning rates, which helps in faster convergence and efficient training, particularly with complex deep learning models. Adam is effective at handling varying gradients during training. To reduce overfitting, **dropout** and L2 regularization are employed to encourage the model to generalize better by preventing it from becoming too reliant on any specific feature.

**Fig 7:**
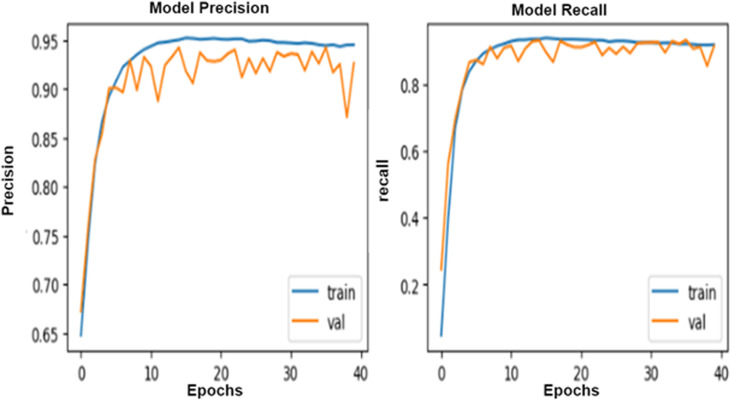
The Roc for the training process of the proposed ResNet 50 with the InceptionV3 algorithm.

**Fig 8:**
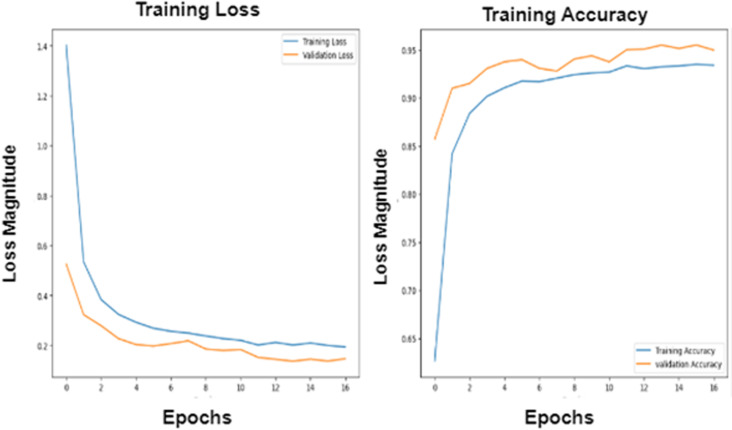
The Roc for the training process of the proposed MobileNet-1 algorithm.

**Fig 9:**
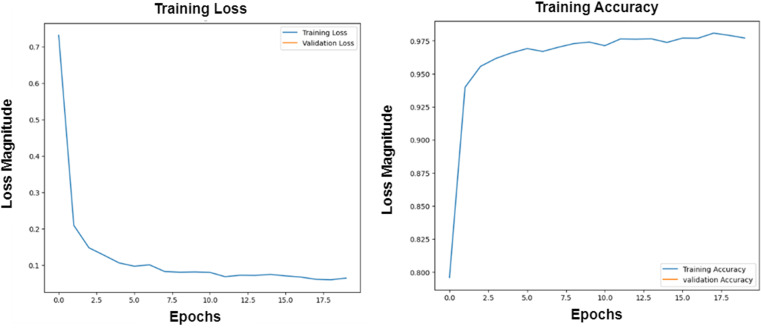
The Roc for the training process of the proposed MobileNet-2 algorithm.

**Fig 10:**
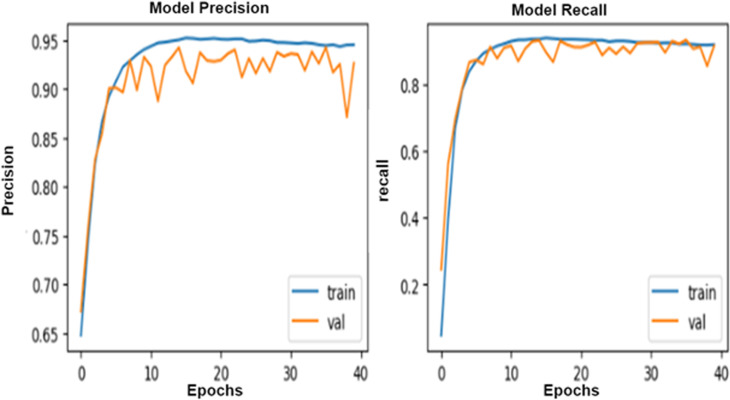
The Roc for the training process of the CNN algorithm.

These hyperparameters and optimization choices are designed to ensure efficient training while maximizing model generalization and accuracy on new data. This can be introduced in Figs 7–10.

## VI. Model deployment

The main objective of this work is to design and implement an integrated framework for monitoring and management of agricultural systems. The following steps illustrate deploying the proposed deep learning model with an application and uploading it to a Railway server while creating an API with Flask.

1. **Preparing the Model:** Ensure that the deep learning model is trained and ready for deployment. This includes saving the trained model weights, architecture, and any necessary pre-processing steps.
2. **Setting up the Application**: Create a web application structure that will serve as the interface for the model. This typically includes files for handling HTTP requests, HTML templates for rendering the user interface, and static files like CSS and JavaScript for styling and interactivity.
3. **Flask API Development**: Use Flask, a Python micro-framework, to create a RESTful API that will expose endpoints for interacting with the deep learning model. Define routes that correspond to different functionalities of the model, such as predicting outcomes or providing status updates.
4. **Integration with the Model**: Integrate the proposed deep learning model into the Flask application. This involves loading the model from disk, initializing it, and defining functions or methods to make predictions based on input data received through API requests.
5. **Testing Locally**: Before deploying to a server, the Flask application has been tested locally to ensure that it functions as expected using the Postman tool and curl to send HTTP requests to the API endpoints and verify their response.
6. **Setting up Railway Server:** Sign up for an account on Railway and create a new project. Follow the instructions provided by Railway to set up the project and configure the deployment environment.
7. **Deploying to Railway:** Once the project is set up, use Railway’s deployment tools to upload the Flask application and any associated files. The Railway will handle the deployment process, including setting up the server environment and managing dependencies.
8. **Accessing the API:** Once deployed, the Flask API will be accessible via a public URL provided by Railway. This URL can be used to send requests to the API from other applications or services.
9. **Monitoring and Maintenance:** Monitor the performance of the deployed application and make any necessary updates or improvements over time. Railway provides tools for monitoring server metrics and managing deployed applications.

**On AI screen,** the capability to capture images using the device’s camera or select photos from a gallery to send to the artificial intelligence system is available. The AI analyzes these images to assess the health status of the plants, identify any diseases present, and recommend the appropriate treatments.

Upon submission, some details results and information about the plant is presented, including its name, current condition, and recommended actions. Whether the plant is suffering from disease or simply needs fertilizers, the proposed AI model provides valuable insights to help farmers maintain their farms.

## VII. Practical implementations

**Fig 11:**
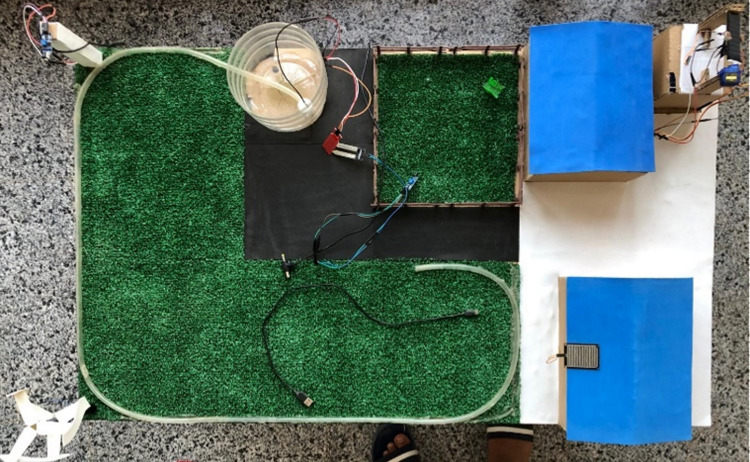
The implemented prototype for the proposed integrated IoT system.

This practical implementation utilizes a smart IoT integrated system to enhance sustainable agriculture by automating key processes. Soil moisture sensor monitors water levels, ensuring optimal irrigation while conserving resources. Rain-drop sensor triggers irrigation only when needed, and the heat sensor works alongside fans to maintain ideal temperature conditions. Fire detection systems safeguard crops from potential hazards, while a water pump automates irrigation processes. The ESP32 serves as the central controller, enabling seamless communication among all devices. This comprehensive setup enhances crop productivity, reduces waste, and promotes sustainable farming practices. The implemented prototype can be shown in Fig 11.

## VIII. Conclusion

In conclusion, this research outlines a comprehensive integrated IoT system for smart agriculture that aims to revolutionize farming practices. By employing innovative technologies to monitor plant health, detect diseases, and recommend tailored solutions for pesticides and fertilizers, the system enhances productivity and crop quality. Additionally, smart irrigation solutions promote efficient water usage, while continuous weather and temperature monitoring ensures that farmers can respond proactively to environmental changes. Accessible through a dedicated mobile application, this integrated approach empowers farmers, agricultural engineers, and veterinarians, facilitating effective management and ultimately leading to increased agricultural productivity and sustainability. The proposed deep learning algorithm has been evaluated and introduced excellent performance for training 38 different types of plant diseases and achieved an accuracy of 92.9% and precision of 92.98%. The deployed mobile application has been tested locally to verify its response using the Postman tool. In future work, we plan to enhance the proposed intelligent framework by integrating IoT-based smart irrigation management systems to improve water resource efficiency and agricultural sustainability [32]. Additionally, we aim to incorporate renewable energy sources and precision robotics to optimize energy consumption and automate field operations [33]. The framework can also be extended to include intelligent food waste management strategies to promote sustainable agriculture [34]. Furthermore, advanced machine learning techniques such as hybrid stacked models and feature selection approaches will be explored to improve classification accuracy and decision-making efficiency, following recent studies in water potability classification and food consumption forecasting [35,36].
